# *In situ* deca­rbonylation of *N*,*N*-di­methyl­formamide to form di­methyl­ammonium cations in the hybrid framework compound {[(CH_3_)_2_NH_2_]_2_[Zn{O_3_PC_6_H_2_(OH)_2_PO_3_}]}_*n*_

**DOI:** 10.1107/S2056989019012969

**Published:** 2019-09-27

**Authors:** Josemaria S. Soriano, Bryan E. Galeas, Paul Garrett, Ryan A. Flores, Juan L. Pinedo, Tsuyoshi A. Kohlgruber, Daniel Felton, Pius O. Adelani

**Affiliations:** a Department of Chemistry and Biochemistry, St. Mary’s University, San Antonio, Texas 78228, USA; b Department of Civil and Environmental Engineering and Earth Sciences, University of Notre Dame, Notre Dame, Indiana 46556, USA; c Department of Chemistry and Biochemistry, University of Notre Dame, Notre Dame, Indiana 46556, USA

**Keywords:** crystal structure, deca­rbonylation, phospho­nic acid, inorganic–organic hybrid framework, hydrogen bonding

## Abstract

The title phospho­nate-based organic–inorganic hybrid framework, poly[bis(dimethylammonium) [(μ_4_-2,5-dihydroxybenzene-1,4-diphosphonato)zinc(II)]], {(C_2_H_8_N)_2_[Zn(C_6_H_4_O_8_P_2_)]}_*n*_, was formed unexpectedly when di­methyl­ammonium cations were formed from the *in situ* deca­rbonylation of the *N*,*N*-di­methyl­formamide solvent. The framework is built up from ZnO_4_ tetra­hedra and bridging di­phospho­nate tetra-anions to generate a three-dimensional network comprising [100] channels occupied by the (CH_3_)_2_NH_2_^+^ cations. Within the channels, an array of N—H⋯O hydrogen bonds help to establish the structure. In addition, intra­molecular O—H⋯O hydrogen bonds between the appended –OH groups of the phenyl ring and adjacent PO_3_^2−^ groups are observed.

## Abstract 2

In the title hybrid organic–inorganic compound, the (CH_3_)_2_NH_2_^+^ cations inter­act with the zinc–phospho­nate framework *via* N—H⋯O hydrogen bonds. The (CH_3_)_2_NH_2_^+^ cations were formed by the *in situ* deca­rbonylation of the *N*,*N*-di­methyl­formamide (DMF) solvent.

## Chemical context   

Studies on the structural chemistry of metal phospho­nates developed as a result of the versatility of the phospho­nate ligands (Zubieta *et al.*, 2011 ▸; Mao, 2007 ▸; Clearfield, 1996 ▸, 1998 ▸, 2002 ▸). A slight modification of the organic residues of the phospho­nic acids (*R*-PO_3_H_2_, where *R* = organic residue) can lead to rich structural diversity. In general, phospho­nates tend to assume various coordination modes as a result of the three coordinating oxygen atoms of the central phospho­rus units. As a consequence, most metal phospho­nates form a low-dimensional and dense layered structure (Deria *et al.*, 2015 ▸; Gagnon *et al.*, 2012 ▸). Nevertheless, a large number of isolated metal phospho­nates have shown various potential applications in ion-exchange, ionic conductivity, gas storage, catalysis, and as small mol­ecule sensors and magnetic inter­actions (Adelani & Albrecht-Schmitt, 2010 ▸; Ramaswamy *et al.*, 2015 ▸; Deria *et al.*, 2015 ▸; Kirumakki *et al.*, 2008 ▸; Brousseau *et al.*, 1997 ▸; Zheng *et al.*, 2011 ▸).

The majority of metal–organic frameworks (MOFs) are designed with carboxyl­ate- and nitro­gen-containing heterocyclic ligands, while phospho­nate-based MOFs are less well studied. One possible explanation may have to do with the predisposition of phospho­nates to precipitate rapidly into less ordered insoluble phases. However, carboxyl­ate-based MOFs are less stable in air and water, and this poses a significant problem if they are to be used in industrial applications. Metal carboxyl­ate MOFs are subject to hydrolysis and are quite soluble in acidic solutions. On the contrary, phospho­nates manifest stronger inter­actions with oxophilic metal ions than carboxyl­ates and are not subject to hydrolysis (Deria *et al.*, 2015 ▸; Gagnon *et al.*, 2012 ▸).

About a decade ago, a crystalline and porous zinc di­phospho­nate MOF, {[Zn(DHBP)](DMF)_2_} (DMF = *N*,*N*-di­methyl­formamide) was reported (Liang & Shimizu, 2007 ▸). These researchers utilized a modified phospho­nate ligand, 1,4-dihy­droxy-2,5-benzene­diphospho­nate (DHBP), to cross-link one-dimensional Zn(*R*PO_3_) columns into an ordered three-dimensional network. Herein, we report the synthesis and structure of the title inorganic–organic hybrid framework, (I), using 1,4-dihy­droxy-2,5-benzene­diphospho­nate *via* the in situ formation of the guest cation.
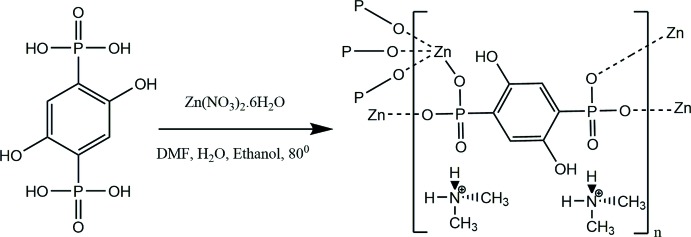


## Structural commentary   

**Figure 1:**
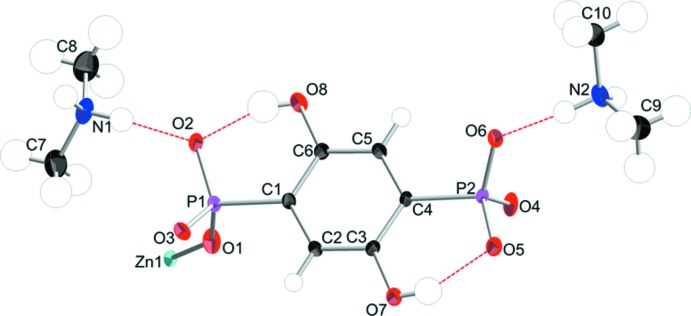
The asymmetric unit of (I) in position 1 − *x*, 1 − *y*, 1 − *z* showing 50% displacement ellipsoids.

**Figure 2:**
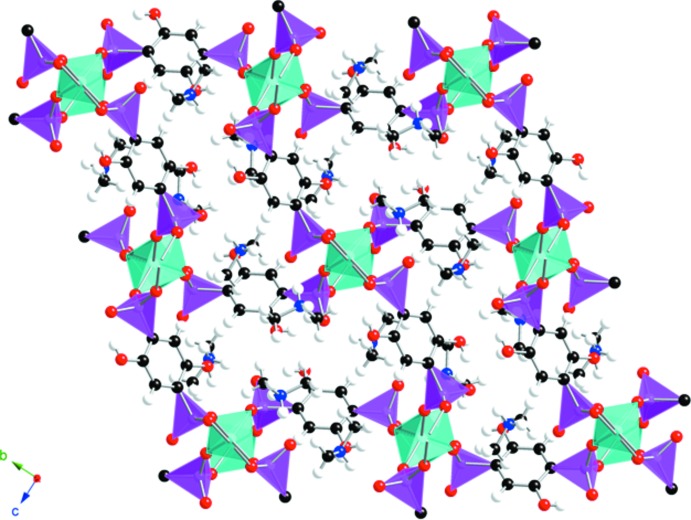
View down [100] of the three-dimensional framework structure of (I) with the ZnO_4_ and PO_3_C moieties shown as polyhedra. Color key: ZnO_4_ groups = cyan, PO_3_C groups = magenta, oxygen = red, carbon = black, hydrogen = white. The (CH_3_)_2_NH_2_^+^ cations are omitted for clarity.

The structure of (I) crystallizes in the monoclinic space group *P*2_1_/*n*. The asymmetric unit contains one Zn^2+^ cation, a C_6_H_4_P_2_O_8_^4−^ hy­droxy­phospho­nate tetra-anion and two (CH_3_)_2_NH_2_^+^ cations (Fig. 1 ▸). The extended structure is constructed from tetra­hedral ZnO_4_ units with the O atoms arising from four rigid phenyl spacers into a three-dimensional framework (Fig. 2 ▸). Two of the oxygen atoms of each PO_3_^2−^ moiety are involved in coordination to the Zn^2+^ ion and the others (O2 and O6) are not. The Zn—O bond distances range from 1.9055 (11) to 1.9671 (11) Å and the hy­droxy­phospho­nate ligand is present in (I) with P—O bonds that range from 1.5129 (11) to 1.5337 (11) Å in length. The latter bond lengths are within the expected range for deprotonated P—O bonds (Liang & Shimizu, 2007 ▸).

The structure of (I) is similar to that of {[Zn(DHBP)](DMF)_2_} (Liang & Shimizu, 2007 ▸; CCDC refcode JIVFUQ) in that the zinc–phospho­nate framework comprises one-dimensional channels occupied by guest species, but with the significant difference that the guest species in JIVFUQ are neutral DMF mol­ecules and the phospho­nate groups are singly, rather than doubly deprotonated to form C_6_H_6_P_2_O_8_^2−^ dianions.

The channels reported here are smaller than those in JIVFUQ and measure approximately 12.9 × 7.1 Å between phenyl groups and 9.9 Å between Zn centers. The (CH_3_)_2_NH_2_^+^ cations in (I) have been formed by the *in situ* deca­rbonylation of the DMF solvent. It is known that *N*,*N*-di­methyl­formamide can undergo loss of CO to form di­methyl­amine in the presence of a metal catalyst or through slow decomposition at elevated temperature around 427 K (Hulushe *et al.*, 2016 ▸; Siddiqui *et al.*, 2012 ▸; Chen *et al.*, 2007 ▸; Karpova *et al.*, 2004 ▸). In the previous reports, the nitrate salts of Mg^2+^/Pb^2+^/Ho^3+^ and chloride salts of Nd^3+^/Zr^4+^ were suggested to act as a metal catalyst in the deca­rbonylation of the DMF solvent.

## Supra­molecular features   

The C6—O8H and C3—O7H groups appended on the phenyl ring of the ligand form intra­molecular O—H⋯O hydrogen bonds with the adjacent *R*PO_3_^2−^ moieties (Figs. 1 ▸ and 3 ▸). Within the channels, the (CH_3_)_2_NH_2_^+^ cations are linked by N—H⋯O hydrogen bonds to the *R*PO_3_^2−^ groups of the framework (Table 1 ▸). Some short C—H⋯O contacts (Table 1 ▸) may help to consolidate the structure.

## Synthesis and crystallization   

The title compound was synthesized by placing Zn(NO_3_)_2_·6H_2_O (29.7 mg, 0.1 mmol) and 2,5-dihy­droxy-1,4-benzene­diphospho­nic acid (27.0 mg, 0.1 mmol) into a 125 ml PTFE-lined Parr reaction vessel along with DMF/H_2_O/ethanol (2.0/0.5/0.5 ml, respectively). The vessel was heated in a programmable furnace at 353 K for 3 d, and then the autoclave was cooled to 296 K at an average rate of 274 K h^−1^. The mother liquor was deca­nted from the products and then placed in a petri dish. The solid products were washed with distilled water, dispersed with ethanol and allowed to dry in air. Colorless tablets of the title compound were isolated and studied for single-crystal X-ray diffraction.

## Refinement   

Crystal data, data collection and structure refinement details are summarized in Table 2 ▸.

## Supplementary Material

Crystal structure: contains datablock(s) I. DOI: 10.1107/S2056989019012969/hb7847sup1.cif

Structure factors: contains datablock(s) I. DOI: 10.1107/S2056989019012969/hb7847Isup2.hkl

CCDC reference: 1954737

Additional supporting information:  crystallographic information; 3D view; checkCIF report

## Figures and Tables

**Figure 3:**
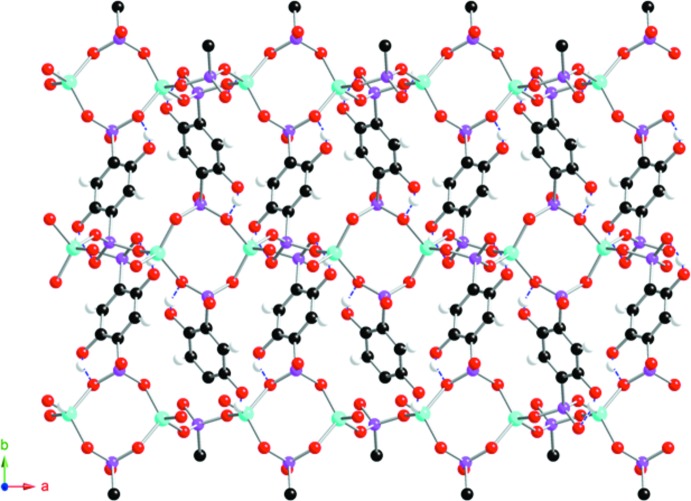
Ball-and-stick representation of the structure of (I) viewed along the [001] axis. The hydrogen bonds involving the –OH groups are drawn as blue dashed lines. Color key as in Fig. 2 ▸.

**Table 1:** Hydrogen-bond geometry (Å, °)

*D*—H⋯*A*	*D*—H	H⋯*A*	*D*⋯*A*	*D*—H⋯*A*
O7—H7*A*⋯O5	0.79 (2)	1.91 (2)	2.6510 (17)	156 (3)
O8—H8*A*⋯O2	0.87 (3)	1.73 (3)	2.5846 (18)	168 (3)
N1—H1*A*⋯O2	0.89 (2)	1.88 (2)	2.7168 (19)	155.2 (18)
N1—H1*B*⋯O6^i^	0.89 (2)	2.02 (2)	2.8125 (19)	148.3 (18)
N2—H2*B*⋯O3^ii^	0.83 (3)	2.07 (3)	2.8558 (19)	158 (2)
N2—H2*C*⋯O6	1.03 (2)	1.63 (2)	2.6518 (18)	173 (2)
C7—H7*C*⋯O4^iii^	0.91 (2)	2.54 (2)	3.443 (3)	174 (2)
C9—H9*B*⋯O8^iv^	1.03 (3)	2.57 (2)	3.445 (3)	142.6 (19)
C10—H10*A*⋯O8^iv^	0.92 (3)	2.42 (3)	3.236 (3)	148 (3)

Symmetry codes: (i) 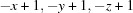; (ii) 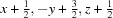; (iii) 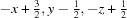; (iv) 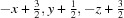.

**Table 2:** Experimental details

Crystal data	
Chemical formula	(C_2_H_8_N)_2_[Zn(C_6_H_4_O_8_P_2_)]
*M*_r_	423.59
Crystal system, space group	Monoclinic, *P*2_1_/*n*
Temperature (K)	220
*a*, *b*, *c* (Å)	8.8455 (5), 16.4492 (9), 11.2721 (6)
β (°)	97.338 (1)
*V* (Å^3^)	1626.67 (15)
*Z*	4
Radiation type	Mo *K*α
μ (mm^−1^)	1.75
Crystal size (mm)	0.09 × 0.03 × 0.03

Data collection	
Diffractometer	Bruker APEXII
Absorption correction	Multi-scan (*SADABS*; Krause *et al.*, 2015 ▸)
*T*_min_, *T*_max_	0.706, 0.746
No. of measured, independent and observed [*I* > 2σ(*I*)] reflections	19692, 4040, 3582
*R*_int_	0.027
(sin θ/λ)_max_ (Å^−1^)	0.681

Refinement	
*R*[*F* ^2^ > 2σ(*F* ^2^)], *wR*(*F* ^2^), *S*	0.022, 0.060, 1.05
No. of reflections	4040
No. of parameters	288
No. of restraints	1
H-atom treatment	All H-atom parameters refined
Δρ_max_, Δρ_min_ (e Å^−3^)	0.42, −0.31

Computer programs: *APEX3* and *SAINT* (Bruker, 2015 ▸), *SHELXT2014/2* (Sheldrick, 2015*a* ▸), *SHELXL2016/6* (Sheldrick, 2015*b* ▸), *XP* in *SHELXTL* (Sheldrick, 2008*a* ▸) and *CIFTAB* (Sheldrick, 2008*b* ▸).

## supplementary crystallographic information

### Crystal data

**Table:**

(C_2_H_8_N)_2_[Zn(C_6_H_4_O_8_P_2_)]	*F*(000) = 872
*M**_r_* = 423.59	*D*_x_ = 1.730 Mg m^−^^3^
Monoclinic, *P*2_1_/*n*	Mo *K*α radiation, λ = 0.71073 Å
*a* = 8.8455 (5) Å	Cell parameters from 8723 reflections
*b* = 16.4492 (9) Å	θ = 2.2–28.8°
*c* = 11.2721 (6) Å	µ = 1.75 mm^−^^1^
β = 97.338 (1)°	*T* = 220 K
*V* = 1626.67 (15) Å^3^	Block, colorless
*Z* = 4	0.09 × 0.03 × 0.03 mm

### Data collection

**Table:**

Bruker APEXII diffractometer	4040 independent reflections
Radiation source: Incoatec micro-focus	3582 reflections with *I* > 2σ(*I*)
Detector resolution: 8.33 pixels mm^-1^	*R*_int_ = 0.027
combination of ω and φ–scans	θ_max_ = 29.0°, θ_min_ = 2.2°
Absorption correction: multi-scan (SADABS; Krause *et al.*, 2015)	*h* = −11→11
*T*_min_ = 0.706, *T*_max_ = 0.746	*k* = −22→21
19692 measured reflections	*l* = −14→14

### Refinement

**Table:**

Refinement on *F*^2^	Primary atom site location: dual
Least-squares matrix: full	Secondary atom site location: difference Fourier map
*R*[*F*^2^ > 2σ(*F*^2^)] = 0.022	Hydrogen site location: difference Fourier map
*wR*(*F*^2^) = 0.060	All H-atom parameters refined
*S* = 1.05	*w* = 1/[σ^2^(*F*_o_^2^) + (0.0327*P*)^2^ + 0.4955*P*] where *P* = (*F*_o_^2^ + 2*F*_c_^2^)/3
4040 reflections	(Δ/σ)_max_ = 0.002
288 parameters	Δρ_max_ = 0.42 e Å^−^^3^
1 restraint	Δρ_min_ = −0.31 e Å^−^^3^

### Special details

**Table:**

Geometry. All esds (except the esd in the dihedral angle between two l.s. planes) are estimated using the full covariance matrix. The cell esds are taken into account individually in the estimation of esds in distances, angles and torsion angles; correlations between esds in cell parameters are only used when they are defined by crystal symmetry. An approximate (isotropic) treatment of cell esds is used for estimating esds involving l.s. planes.

### Fractional atomic coordinates and isotropic or equivalent isotropic displacement parameters (Å^2^)

**Table:**

	*x*	*y*	*z*	*U*_iso_*/*U*_eq_	
Zn1	0.73693 (2)	0.50748 (2)	−0.00920 (2)	0.01208 (6)	
P1	0.53496 (4)	0.52463 (2)	0.21300 (3)	0.01348 (9)	
P2	0.52017 (4)	0.86078 (2)	0.50529 (3)	0.01240 (8)	
O1	0.61568 (13)	0.54669 (7)	0.10670 (10)	0.0251 (3)	
O2	0.61879 (14)	0.46378 (7)	0.29864 (11)	0.0241 (3)	
O3	0.37005 (12)	0.49818 (6)	0.17307 (10)	0.0173 (2)	
O4	0.66080 (13)	0.90494 (6)	0.47282 (10)	0.0217 (2)	
O5	0.37382 (13)	0.90513 (7)	0.45507 (10)	0.0220 (2)	
O6	0.53036 (13)	0.84096 (7)	0.63713 (9)	0.0216 (2)	
O7	0.31114 (15)	0.80860 (7)	0.26689 (12)	0.0287 (3)	
O8	0.71416 (17)	0.57148 (8)	0.45775 (13)	0.0382 (4)	
C1	0.52417 (16)	0.61778 (9)	0.29782 (13)	0.0137 (3)	
C2	0.42525 (17)	0.67996 (9)	0.25376 (13)	0.0161 (3)	
C3	0.41615 (17)	0.75253 (9)	0.31601 (13)	0.0155 (3)	
C4	0.51147 (16)	0.76540 (8)	0.42426 (13)	0.0128 (3)	
C5	0.60872 (17)	0.70290 (9)	0.46917 (14)	0.0176 (3)	
C6	0.61546 (17)	0.62969 (9)	0.40804 (14)	0.0186 (3)	
C7	0.5494 (3)	0.29794 (13)	0.13241 (19)	0.0387 (5)	
N1	0.57872 (18)	0.30131 (9)	0.26452 (15)	0.0277 (3)	
C8	0.7362 (3)	0.27776 (15)	0.3120 (2)	0.0443 (5)	
C9	0.5188 (3)	1.01513 (14)	0.8070 (2)	0.0356 (4)	
N2	0.63088 (18)	0.94927 (9)	0.80290 (13)	0.0246 (3)	
C10	0.6615 (3)	0.90359 (13)	0.91563 (18)	0.0371 (5)	
H1A	0.568 (2)	0.3527 (14)	0.287 (2)	0.038 (6)*	
H1B	0.515 (2)	0.2695 (14)	0.2980 (19)	0.037 (6)*	
H2A	0.360 (2)	0.6731 (12)	0.1787 (17)	0.026 (5)*	
H2B	0.709 (3)	0.9705 (14)	0.783 (2)	0.041 (6)*	
H2C	0.588 (3)	0.9111 (15)	0.735 (2)	0.054 (7)*	
H5A	0.676 (2)	0.7101 (11)	0.5433 (17)	0.024 (5)*	
H7A	0.306 (3)	0.8427 (16)	0.316 (2)	0.050 (7)*	
H7B	0.450 (3)	0.3113 (17)	0.109 (2)	0.071 (9)*	
H7C	0.620 (2)	0.3284 (16)	0.101 (2)	0.055 (7)*	
H7D	0.566 (3)	0.2430 (15)	0.109 (2)	0.044 (6)*	
H8A	0.691 (3)	0.5308 (18)	0.410 (3)	0.064 (8)*	
H8B	0.806 (3)	0.3177 (15)	0.281 (2)	0.052 (7)*	
H8C	0.750 (3)	0.2805 (15)	0.402 (2)	0.053 (7)*	
H8D	0.748 (3)	0.2232 (16)	0.278 (2)	0.056 (7)*	
H9A	0.501 (2)	1.0337 (13)	0.729 (2)	0.036 (6)*	
H9B	0.569 (3)	1.0562 (16)	0.869 (2)	0.054 (7)*	
H9C	0.431 (3)	0.9924 (13)	0.828 (2)	0.042 (7)*	
H10A	0.719 (4)	0.936 (2)	0.970 (3)	0.088 (11)*	
H10B	0.564 (4)	0.8940 (17)	0.947 (3)	0.077 (9)*	
H10C	0.716 (3)	0.8557 (17)	0.902 (2)	0.062 (8)*	

### Atomic displacement parameters (Å^2^)

**Table:**

	*U*^11^	*U*^22^	*U*^33^	*U*^12^	*U*^13^	*U*^23^
Zn1	0.01243 (9)	0.01005 (9)	0.01414 (9)	−0.00104 (6)	0.00319 (6)	−0.00073 (6)
P1	0.01327 (18)	0.01165 (17)	0.01539 (18)	0.00072 (13)	0.00134 (14)	−0.00453 (14)
P2	0.01391 (18)	0.00907 (17)	0.01421 (18)	0.00096 (13)	0.00174 (14)	−0.00254 (13)
O1	0.0271 (6)	0.0243 (6)	0.0268 (6)	−0.0033 (5)	0.0146 (5)	−0.0084 (5)
O2	0.0287 (6)	0.0141 (5)	0.0268 (6)	0.0053 (5)	−0.0070 (5)	−0.0053 (5)
O3	0.0153 (5)	0.0196 (5)	0.0167 (5)	−0.0027 (4)	0.0006 (4)	−0.0046 (4)
O4	0.0219 (6)	0.0142 (5)	0.0302 (6)	−0.0051 (4)	0.0085 (5)	−0.0057 (4)
O5	0.0210 (6)	0.0185 (5)	0.0253 (6)	0.0094 (4)	−0.0018 (5)	−0.0068 (5)
O6	0.0331 (6)	0.0162 (5)	0.0152 (5)	−0.0024 (5)	0.0023 (5)	−0.0024 (4)
O7	0.0350 (7)	0.0169 (6)	0.0293 (7)	0.0113 (5)	−0.0149 (5)	−0.0073 (5)
O8	0.0463 (8)	0.0237 (7)	0.0367 (8)	0.0212 (6)	−0.0249 (6)	−0.0140 (6)
C1	0.0132 (7)	0.0125 (7)	0.0156 (7)	0.0004 (5)	0.0019 (5)	−0.0032 (5)
C2	0.0177 (7)	0.0144 (7)	0.0152 (7)	−0.0005 (6)	−0.0020 (6)	−0.0025 (6)
C3	0.0158 (7)	0.0123 (6)	0.0176 (7)	0.0023 (5)	−0.0005 (6)	0.0002 (5)
C4	0.0142 (7)	0.0105 (6)	0.0141 (7)	−0.0008 (5)	0.0029 (5)	−0.0016 (5)
C5	0.0184 (7)	0.0160 (7)	0.0168 (7)	0.0018 (6)	−0.0032 (6)	−0.0032 (6)
C6	0.0192 (7)	0.0147 (7)	0.0207 (8)	0.0062 (6)	−0.0028 (6)	−0.0032 (6)
C7	0.0482 (13)	0.0331 (11)	0.0375 (11)	−0.0098 (10)	0.0162 (10)	−0.0045 (9)
N1	0.0312 (8)	0.0175 (7)	0.0373 (9)	−0.0035 (6)	0.0154 (7)	−0.0030 (6)
C8	0.0363 (11)	0.0374 (12)	0.0601 (16)	0.0052 (9)	0.0091 (11)	−0.0063 (11)
C9	0.0353 (11)	0.0405 (11)	0.0326 (11)	0.0034 (9)	0.0096 (9)	0.0022 (9)
N2	0.0254 (8)	0.0297 (8)	0.0202 (7)	−0.0096 (6)	0.0087 (6)	−0.0050 (6)
C10	0.0577 (14)	0.0300 (10)	0.0236 (9)	0.0000 (10)	0.0052 (9)	−0.0045 (8)

### Geometric parameters (Å, º)

**Table:**

Zn1—O1	1.9055 (11)	C5—C6	1.392 (2)
Zn1—O3^i^	1.9671 (11)	C5—H5A	0.971 (19)
Zn1—O4^ii^	1.9330 (11)	C7—N1	1.480 (3)
Zn1—O5^iii^	1.9543 (10)	C7—H7B	0.92 (2)
P1—O1	1.5151 (12)	C7—H7C	0.91 (2)
P1—O2	1.5169 (12)	C7—H7D	0.96 (2)
P1—O3	1.5337 (11)	N1—C8	1.479 (3)
P1—C1	1.8150 (14)	N1—H1A	0.89 (2)
P2—O6	1.5129 (11)	N1—H1B	0.89 (2)
P2—O4	1.5249 (11)	C8—H8B	1.00 (3)
P2—O5	1.5301 (11)	C8—H8C	1.01 (3)
P2—C4	1.8121 (14)	C8—H8D	0.98 (3)
O7—C3	1.3743 (18)	C9—N2	1.473 (3)
O7—H7A	0.79 (3)	C9—H9A	0.92 (2)
O8—C6	1.3668 (19)	C9—H9B	1.03 (3)
O8—H8A	0.86 (3)	C9—H9C	0.92 (3)
C1—C2	1.395 (2)	N2—C10	1.471 (2)
C1—C6	1.406 (2)	N2—H2B	0.83 (2)
C2—C3	1.392 (2)	N2—H2C	1.02 (3)
C2—H2A	0.968 (19)	C10—H10A	0.92 (4)
C3—C4	1.408 (2)	C10—H10B	0.98 (3)
C4—C5	1.394 (2)	C10—H10C	0.95 (3)
			
O1—Zn1—O4^ii^	116.04 (5)	O8—C6—C5	117.94 (14)
O1—Zn1—O5^iii^	108.06 (5)	O8—C6—C1	121.95 (13)
O4^ii^—Zn1—O5^iii^	113.58 (5)	C5—C6—C1	120.11 (13)
O1—Zn1—O3^i^	114.48 (5)	N1—C7—H7B	108.8 (17)
O4^ii^—Zn1—O3^i^	108.30 (5)	N1—C7—H7C	109.5 (15)
O5^iii^—Zn1—O3^i^	94.45 (4)	H7B—C7—H7C	116 (2)
O1—P1—O2	114.83 (7)	N1—C7—H7D	107.5 (14)
O1—P1—O3	111.25 (7)	H7B—C7—H7D	109 (2)
O2—P1—O3	111.65 (7)	H7C—C7—H7D	106 (2)
O1—P1—C1	106.03 (7)	C8—N1—C7	112.97 (17)
O2—P1—C1	106.03 (7)	C8—N1—H1A	106.0 (14)
O3—P1—C1	106.38 (6)	C7—N1—H1A	107.9 (14)
O6—P2—O4	112.98 (7)	C8—N1—H1B	108.3 (14)
O6—P2—O5	114.05 (7)	C7—N1—H1B	111.4 (14)
O4—P2—O5	111.20 (7)	H1A—N1—H1B	110 (2)
O6—P2—C4	107.57 (6)	N1—C8—H8B	107.3 (14)
O4—P2—C4	105.95 (6)	N1—C8—H8C	110.0 (14)
O5—P2—C4	104.29 (6)	H8B—C8—H8C	109 (2)
P1—O1—Zn1	145.53 (8)	N1—C8—H8D	104.0 (15)
P1—O3—Zn1^i^	127.91 (7)	H8B—C8—H8D	111 (2)
P2—O4—Zn1^iv^	137.62 (7)	H8C—C8—H8D	115 (2)
P2—O5—Zn1^v^	142.34 (7)	N2—C9—H9A	104.4 (14)
C3—O7—H7A	107.0 (18)	N2—C9—H9B	105.8 (14)
C6—O8—H8A	101.6 (19)	H9A—C9—H9B	116 (2)
C2—C1—C6	118.38 (13)	N2—C9—H9C	107.6 (14)
C2—C1—P1	120.27 (11)	H9A—C9—H9C	109 (2)
C6—C1—P1	121.34 (11)	H9B—C9—H9C	113 (2)
C3—C2—C1	121.54 (14)	C10—N2—C9	113.51 (16)
C3—C2—H2A	118.3 (11)	C10—N2—H2B	112.3 (17)
C1—C2—H2A	120.1 (11)	C9—N2—H2B	106.7 (16)
O7—C3—C2	116.88 (13)	C10—N2—H2C	110.0 (14)
O7—C3—C4	123.19 (13)	C9—N2—H2C	106.8 (14)
C2—C3—C4	119.93 (13)	H2B—N2—H2C	107 (2)
C5—C4—C3	118.52 (13)	N2—C10—H10A	108 (2)
C5—C4—P2	118.11 (11)	N2—C10—H10B	108.4 (17)
C3—C4—P2	123.32 (11)	H10A—C10—H10B	107 (2)
C6—C5—C4	121.45 (14)	N2—C10—H10C	109.3 (16)
C6—C5—H5A	118.1 (11)	H10A—C10—H10C	110 (3)
C4—C5—H5A	120.4 (11)	H10B—C10—H10C	114 (2)
			
O2—P1—O1—Zn1	37.88 (16)	C1—C2—C3—O7	−177.72 (14)
O3—P1—O1—Zn1	−90.15 (14)	C1—C2—C3—C4	2.0 (2)
C1—P1—O1—Zn1	154.59 (13)	O7—C3—C4—C5	176.85 (14)
O1—P1—O3—Zn1^i^	−0.87 (10)	C2—C3—C4—C5	−2.8 (2)
O2—P1—O3—Zn1^i^	−130.59 (8)	O7—C3—C4—P2	−5.8 (2)
C1—P1—O3—Zn1^i^	114.17 (8)	C2—C3—C4—P2	174.51 (11)
O6—P2—O4—Zn1^iv^	−63.56 (12)	O6—P2—C4—C5	−43.03 (13)
O5—P2—O4—Zn1^iv^	66.18 (12)	O4—P2—C4—C5	78.06 (13)
C4—P2—O4—Zn1^iv^	178.91 (10)	O5—P2—C4—C5	−164.49 (12)
O6—P2—O5—Zn1^v^	43.33 (14)	O6—P2—C4—C3	139.65 (13)
O4—P2—O5—Zn1^v^	−85.85 (13)	O4—P2—C4—C3	−99.26 (13)
C4—P2—O5—Zn1^v^	160.39 (11)	O5—P2—C4—C3	18.19 (14)
O1—P1—C1—C2	71.83 (13)	C3—C4—C5—C6	1.5 (2)
O2—P1—C1—C2	−165.68 (12)	P2—C4—C5—C6	−175.95 (12)
O3—P1—C1—C2	−46.70 (14)	C4—C5—C6—O8	179.76 (15)
O1—P1—C1—C6	−107.45 (13)	C4—C5—C6—C1	0.7 (2)
O2—P1—C1—C6	15.04 (15)	C2—C1—C6—O8	179.40 (15)
O3—P1—C1—C6	134.02 (13)	P1—C1—C6—O8	−1.3 (2)
C6—C1—C2—C3	0.2 (2)	C2—C1—C6—C5	−1.6 (2)
P1—C1—C2—C3	−179.06 (12)	P1—C1—C6—C5	177.73 (12)

Symmetry codes: (i) −*x*+1, −*y*+1, −*z*; (ii) −*x*+3/2, *y*−1/2, −*z*+1/2; (iii) *x*+1/2, −*y*+3/2, *z*−1/2; (iv) −*x*+3/2, *y*+1/2, −*z*+1/2; (v) *x*−1/2, −*y*+3/2, *z*+1/2.

### Hydrogen-bond geometry (Å, º)

**Table:**

*D*—H···*A*	*D*—H	H···*A*	*D*···*A*	*D*—H···*A*
O7—H7*A*···O5	0.79 (2)	1.91 (2)	2.6510 (17)	156 (3)
O8—H8*A*···O2	0.87 (3)	1.73 (3)	2.5846 (18)	168 (3)
N1—H1*A*···O2	0.89 (2)	1.88 (2)	2.7168 (19)	155.2 (18)
N1—H1*B*···O6^vi^	0.89 (2)	2.02 (2)	2.8125 (19)	148.3 (18)
N2—H2*B*···O3^vii^	0.83 (3)	2.07 (3)	2.8558 (19)	158 (2)
N2—H2*C*···O6	1.03 (2)	1.63 (2)	2.6518 (18)	173 (2)
C7—H7*C*···O4^ii^	0.91 (2)	2.54 (2)	3.443 (3)	174 (2)
C9—H9*B*···O8^viii^	1.03 (3)	2.57 (2)	3.445 (3)	142.6 (19)
C10—H10*A*···O8^viii^	0.92 (3)	2.42 (3)	3.236 (3)	148 (3)

Symmetry codes: (ii) −*x*+3/2, *y*−1/2, −*z*+1/2; (vi) −*x*+1, −*y*+1, −*z*+1; (vii) *x*+1/2, −*y*+3/2, *z*+1/2; (viii) −*x*+3/2, *y*+1/2, −*z*+3/2.
